# TREM2 in age-related macular degeneration: a microglia-centered perspective in the retinal myeloid landscape

**DOI:** 10.3389/fopht.2026.1804578

**Published:** 2026-04-17

**Authors:** Shengyu Zhu, Taoshuo Yang, Limei Sheng, Lei Shi

**Affiliations:** 1 Ophthalmology, Danyang Hospital of Traditional Chinese Medicine, Zhenjiang, Jiangsu, China; 2 College of Medical, Veterinary & Life Sciences, University of Glasgow, Scotland, United Kingdom; 3 College of Physical Education, Yangzhou University, Yangzhou, Jiangsu, China

**Keywords:** age-related macular degeneration, aging, inflammation, microglia, TREM2

## Abstract

Age-related macular degeneration (AMD) is a leading cause of irreversible central vision loss in older adults. Advanced AMD comprises an atrophic (“dry”) form characterized by retinal pigment epithelium (RPE) and photoreceptor degeneration and a neovascular (“wet”) form driven by choroidal neovascularization (CNV). Beyond genetic predisposition and environmental stressors, chronic dysregulation of innate immunity is increasingly recognized as a convergent mechanism linking drusen/Bruch’s membrane alterations to outer retinal cell death and pathological angiogenesis. Retinal myeloid cells—including resident microglia and, in specific disease contexts, recruited monocyte-derived macrophages—can support homeostasis by clearing lipids and cellular debris, yet may also exacerbate inflammation, matrix remodeling, and neovascularization. Triggering receptor expressed on myeloid cells 2 (TREM2) is an innate immune receptor expressed by microglia and other myeloid cells that regulates phagocytosis, lipid handling, migration, survival, immunometabolism, and inflammatory tone. Recent retinal studies suggest that TREM2-associated programs can restrain lesion expansion in outer retinal degeneration models and modulate CNV severity in experimental neovascularization; however, interpretation remains limited by disease stage, anatomical niche, and the difficulty of cleanly separating microglia from infiltrating macrophages *in vivo*. Here, we synthesize current evidence on retinal myeloid contributions to dry and neovascular AMD, provide an updated mechanistic framework for TREM2 signaling, and discuss therapeutic strategies and translational challenges for targeting TREM2 in AMD.

## 1 Introduction

Age-related macular degeneration (AMD) is a chronic, progressive macular disease and the leading cause of blindness in individuals over 65 worldwide (1). Its pathogenesis is multifactorial, involving oxidative stress, lipid metabolism dysfunction, genetic susceptibility, and immune dysregulation (2). Clinically, advanced AMD is categorized into dry (atrophic) and wet (neovascular) forms (3). Dry AMD, accounting for ~85–90% of cases, is marked by drusen accumulation and gradual atrophy of the retinal pigment epithelium (RPE) and photoreceptors, culminating in geographic atrophy (GA). Wet AMD (~10–15% of cases) features aberrant choroidal neovascularization (CNV) with fragile, leaky vessels that cause subretinal fluid and hemorrhages, leading to rapid and severe vision loss.

Microglia are the parenchymal resident macrophages of the retina. Together with other ocular mononuclear phagocytes—including retinal perivascular macrophages, choroidal macrophages, and vitreous hyalocytes—they contribute to immune surveillance, tissue homeostasis, and debris clearance in anatomically distinct ocular niches (3–5). During AMD and related outer retinal degenerations, myeloid cells redistribute toward the subretinal space and RPE–choroid interface and undergo transcriptional reprogramming, with consequences that may be protective (debris clearance and tissue stabilization) or pathological (sustained inflammatory signaling, extracellular matrix remodeling, and promotion of angiogenesis) (6–8).

TREM2(Triggering Receptor Expressed on Myeloid Cells 2) has emerged as a key immunometabolic checkpoint in microglia and other myeloid cells. Notably, in neurodegenerative diseases like Alzheimer’s, TREM2 gene variants alter microglial function and disease risk, underscoring its key role in age-related neuroinflammation (9). In human AMD retina, TREM2 is downregulated in association with miR-34a upregulation, and experimental retinal degeneration studies further indicate that Trem2 supports retinal myeloid migration and phagocytic responses that can limit outer retinal injury (10, 11), suggesting that loss of TREM2 activity can accelerate retinal degeneration. Conversely, augmenting TREM2 signaling enhances microglial migration and phagocytosis in animal models, thereby protecting retinal neurons from damage.

In light of these insights, the present review focuses on how microglial TREM2 contributes to AMD pathogenesis and assesses its potential as a therapeutic target. We synthesize recent findings on TREM2’s roles in regulating retinal inflammation, phagocytic metabolism, and pathological angiogenesis, aiming to clarify how TREM2 shapes AMD progression and to evaluate prospective TREM2-targeted interventions for AMD.

## 2 Age-related macular degeneration

AMD results from progressive degeneration of the macula, ultimately causing central vision loss. Late-stage disease is broadly classified into dry (atrophic) and wet (neovascular) forms, which stem from common early changes (drusen deposits, RPE pigmentary disturbances) but diverge in their end-stage pathology.

### 2.1 Dry age-related macular degeneration

Dry AMD is the more prevalent form (~85–90% of cases). Its hallmarks include drusen—yellow-white extracellular deposits between the RPE and Bruch’s membrane—and gradual degeneration of the macular RPE and photoreceptors, culminating in geographic atrophy (GA) (12). Drusen are composed of lipids (e.g., cholesterol esters, phospholipids), misfolded proteins (complement components, vitronectin, amyloid-β, apolipoproteins), and other metabolic waste products (13, 14). While small drusen can be clinically benign, large confluent drusen mark intermediate AMD and often presage RPE atrophy. As dry AMD advances, patchy RPE cell loss expands into coalescent atrophic areas, and dependent photoreceptors die due to lost support. GA represents the end-stage of dry AMD, yielding an irreversible central scotoma.

Multiple factors contribute to dry AMD pathogenesis. Oxidative stress is a central driver: the macula’s lifetime of intense light exposure and high metabolic activity produces reactive oxygen species that damage RPE macromolecules and organelles (15). Age-related accumulation of lipofuscin (indigestible lipid–protein aggregates) in RPE cells further compromises their function (16). Lipid metabolism dysregulation also plays a key role: the RPE continuously secretes apolipoprotein B/E–containing lipoproteins into Bruch’s membrane as part of retinal lipid recycling, but with age these lipid-rich deposits accumulate and trigger chronic inflammation and complement activation (17, 18). Genetic studies bolster these mechanisms: variants in complement genes (e.g., CFH, C3, C2, CFB, CFI) and lipid-handling genes (e.g., APOE, LIPC, ABCA1, CETP) significantly influence dry AMD risk (17, 19).

**Figure 1:**
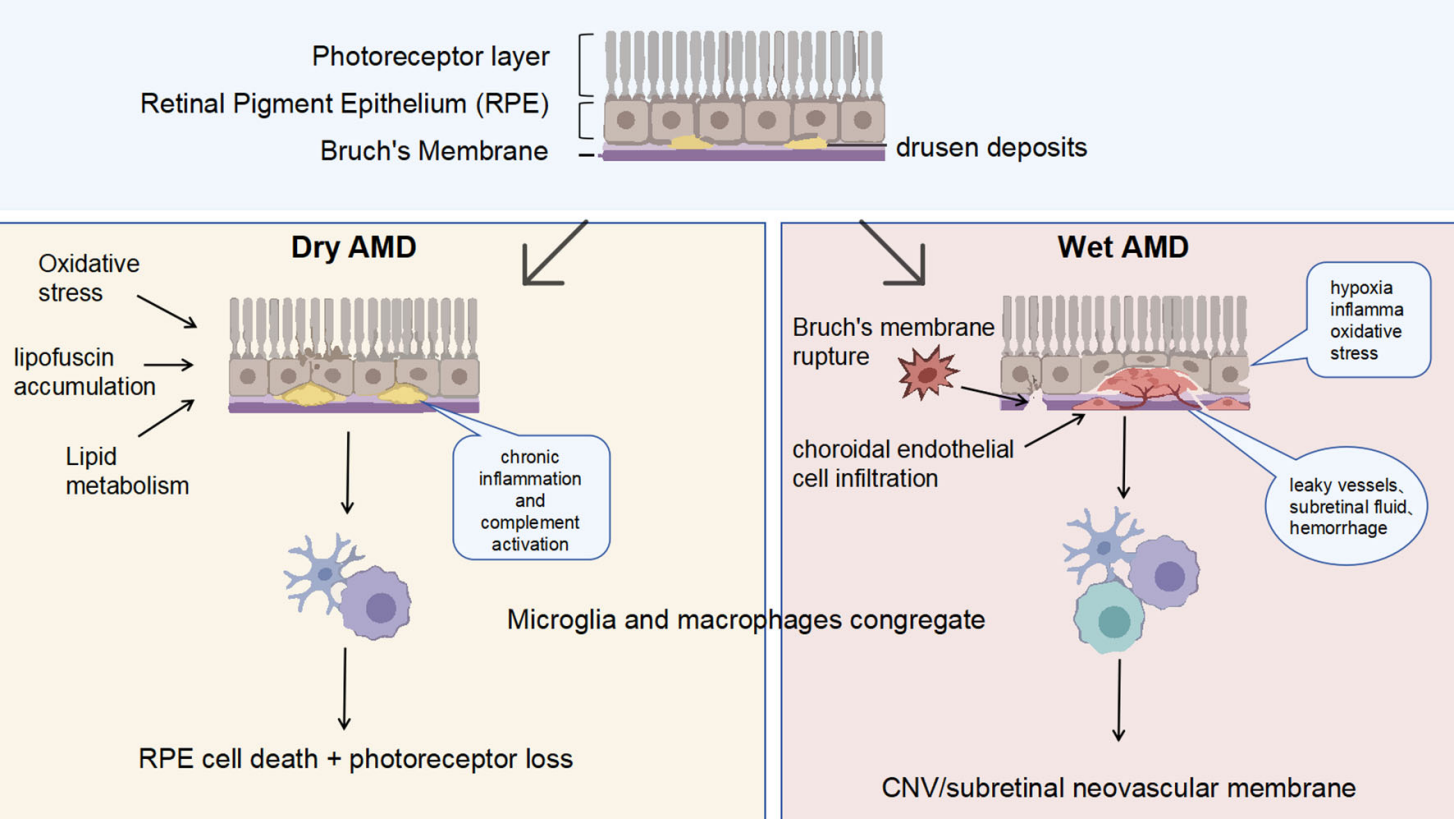
Diagram of AMD pathogenesis. (Dry AMD is characterized by drusen accumulation beneath the RPE, where oxidative stress, lipofuscin buildup, and lipid dysregulation drive complement-associated inflammation, microglial/macrophage accumulation, and progressive RPE and photoreceptor loss. Wet AMD is characterized by VEGF-driven choroidal neovascularization through Bruch’s membrane, in which lesion-associated retinal myeloid cells help sustain an inflammatory angiogenic milieu, leading to fragile leaky vessels, subretinal fluid, and hemorrhage).

Persistent inflammation is another hallmark of dry AMD. Drusen components such as oxidized lipids, complement fragments, and amyloid-β chronically activate innate immunity. The complement cascade often remains upregulated, and stressed RPE cells along with infiltrating immune cells secrete pro-inflammatory cytokines (e.g., IL-6, IL-1β), creating a hostile milieu that accelerates RPE and photoreceptor cell death and impairs retinal integrity. Importantly, outer retinal loss in AMD-related settings is unlikely to be explained by apoptosis alone; multiple regulated cell-death pathways—including necroptosis, inflammasome-associated pyroptosis, and ferroptosis—have been implicated across RPE/photoreceptor injury paradigms (13, 20, 21). Therefore, we use the umbrella term cell death throughout this review unless a specific pathway is directly demonstrated. At sites of drusen accumulation and outer retinal atrophy, microglia/macrophages can engulf lipid-rich debris and dying cells; however, persistent activation may sustain cytokine and complement-associated inflammation and exacerbate RPE and photoreceptor injury (10, 22). Thus, dry AMD often teeters on a tipping point between beneficial debris-clearing inflammation and self-perpetuating, destructive inflammation(A schematic diagram is shown in **Figure 1**).

### 2.2 Wet age-related macular degeneration

Wet AMD (neovascular AMD) is characterized by abnormal choroidal blood vessels growing into the subretinal space. This pathological angiogenesis is driven largely by VEGF signaling within the inflammatory and angiogenic microenvironment of neovascular AMD (23). The neovascular vessels are fragile and leaky, causing subretinal fluid accumulation and hemorrhages. Clinically, wet AMD often manifests as acute or subacute vision loss from exudative retinal detachment or direct photoreceptor damage. Although only ~10–15% of AMD patients develop neovascular lesions, this subtype accounts for the majority of severe vision loss in AMD (24).

Pathologically, wet AMD entails the rupture of Bruch’s membrane and infiltration of choroidal endothelial cells into the retina, forming subretinal (or occasionally intraretinal) neovascular membranes (25). These lesions often arise at sites of large drusen deposits or RPE detachments. In the aged macula, several processes converge to drive angiogenesis: RPE atrophy creates local ischemia that induces VEGF; chronic complement activation and inflammation further upregulate pro-angiogenic signals; and elevated oxidative stress stabilizes HIF-1α, shifting the balance in favor of new vessel growth (26). The outcome is a pathological neovascular network that distorts and damages retinal architecture.

Retinal myeloid cells actively contribute to neovascular AMD pathology, but their effects are best understood in relation to cell of origin, lesion niche, and disease stage rather than within a binary M1/M2 framework (27, 28). In experimental CNV, lesion-associated myeloid cells can amplify angiogenesis through inflammatory cytokines, VEGF-family signaling, extracellular matrix remodeling, and recruitment of additional leukocytes (29–31). At the same time, not all retinal myeloid responses are proangiogenic; some may limit lesion expansion, promote debris clearance, or modulate fibrotic repair. Thus, the functional impact of retinal myeloid cells in CNV is heterogeneous and temporally dynamic(A schematic diagram is shown in **Figure 1**).

## 3 Microglia in age-related macular degeneration

Because AMD is fundamentally an inflammatory neurodegenerative condition, considerable research has centered on microglia. These cells are the resident macrophages of the retina, derived from yolk sac progenitors and maintained by self-renewal in the CNS (31). In a healthy retina, microglia occupy the inner and outer plexiform layers, constantly interacting with neurons, Müller glia, and RPE cells. They provide surveillance and support, for example by phagocytosing shed photoreceptor outer segments and secreting neurotrophic factors (32, 33). In AMD and related outer retinal degeneration models, retinal microglia undergo marked activation, migrate from the plexiform layers toward the subretinal space, and adopt injury-responsive transcriptional states whose functions depend on anatomical niche and disease stage rather than a simple binary polarization scheme (10, 28).

### 3.1 Retinal microglia and monocyte-derived macrophages

Retinal microglia are yolk-sac-derived resident macrophages that normally occupy the plexiform layers and contribute to immune surveillance, synaptic maintenance, and tissue homeostasis (34). In contrast, monocyte-derived macrophages can be recruited under inflammatory, degenerative, or neovascular conditions, particularly in subretinal, perivascular, and lesion-associated niches (35).

Retinal microglia share several pan-myeloid markers with other macrophage-lineage cells, especially under disease conditions. For example, IBA1/AIF1 and CD68 are not microglia-specific, whereas P2RY12 and TMEM119 are more informative for homeostatic microglia but can be downregulated during injury (28, 36). Moreover, TREM2 is not microglia-exclusive and can be expressed by recruited monocyte-derived macrophages depending on context. Therefore, distinguishing microglia from infiltrating macrophages in AMD lesions requires a multi-marker strategy and, ideally, lineage tracing or microglia-restricted genetic tools (37).

These issues have important interpretive consequences for AMD research. Conclusions specifically about “microglial TREM2” should ideally be reserved for lineage-tracing studies, microglia-selective genetic tools, or convergent marker strategies (31, 38, 39). By contrast, data derived from whole-retina transcriptomics, pharmacologic agonists, global knockouts, or lesion-wide immunostaining are more appropriately interpreted as reflecting retinal myeloid cells or mononuclear phagocytes more broadly.

With aging and in AMD, microglia frequently shift into an activated state. Activated microglia assume an amoeboid morphology, upregulate markers such as CD68 and MHC-II, and secrete inflammatory mediators. A hallmark of early AMD is the migration of microglia from the inner retina into the subretinal space (beneath photoreceptors and above the RPE) (28, 40). These subretinal microglia likely reflects efforts to clear debris (e.g., drusen and dying cells), but it also signifies chronic inflammation. Not all macrophages in AMD lesions originate as resident microglia; circulating monocytes are recruited via chemokine signals (like CCL2) and enter the retina through the vasculature (41, 42). The CCL2–CCR2 axis is critical for this peripheral recruitment, and blocking it can attenuate pathological subretinal inflammation (43).

Single-cell RNA sequencing of retinas from AMD patients and models reveals heterogeneous microglial activation states. Pro-inflammatory “M1-like” microglia accumulate in AMD, marked by high expression of IL1B, TNF, IL6 and suppression of homeostatic genes like CX3CR1 (13). Conversely, a subset of microglia acquires a damage-associated microglia (DAM) phenotype—reminiscent of those in other neurodegenerative diseases—characterized by upregulation of phagocytosis and lipid metabolism genes such as TREM2, APOE, and LGALS3 (galectin-3) (44, 45). A recent study identified a “galectin-3-positive” microglial subset accumulating at sites of atrophy in AMD (10). Thus, microglia in AMD are not a uniform population; their effects vary by subset and activation program. Key cues shaping microglial activation include damage-associated molecular patterns from stressed cells (ATP, DNA, oxidized lipids), the cytokine milieu (e.g., IFN-γ vs. IL-4), and intrinsic regulators like TREM2.

### 3.2 Neuroinflammatory environment in AMD

Aging tissues, including the retina, develop a low-grade “para-inflammatory” state as they accumulate stress. In AMD, this baseline inflammation becomes amplified and maladaptive (5). Stressed retinal cells (RPE and neurons) chronically release pro-inflammatory signals—such as IL-1β, IL-6, IL-8, TNF-α, CCL2, CX3CL1, and colony-stimulating factors (46, 47), which together establish a neuroinflammatory milieu. This cocktail of cytokines and chemokines activates resident microglia and attracts peripheral myeloid cells into the retina (22).

Once activated, subretinal myeloid cells may adopt inflammatory, interferon-responsive, lipid-associated, or galectin-3-positive injury-responsive programs rather than discrete M1/M2 states (10, 48). Their effector output includes cytokine release, complement modulation, phagocytosis, metabolic remodeling, and crosstalk with RPE cells, Müller glia, endothelial cells, and recruited neutrophils (48). In early degeneration, insufficient clearance of dying cells and lipid-rich debris may intensify inflammation; conversely, appropriately localized injury-responsive programs may help contain lesion progression by promoting tissue cleanup and inflammatory restraint (46, 47).

Despite these pro-inflammatory tendencies, microglia retain protective capacities even in inflamed conditions. They secrete anti-inflammatory cytokines (e.g., IL-10, TGF-β) in attempts to resolve inflammation (49, 50). Crucially, microglia also phagocytose dead and dying cells; without prompt clearance, cellular corpses release toxic contents that exacerbate tissue injury. Growing evidence suggests that when microglia are appropriately activated (e.g., into a DAM-like state), they can slow retinal degeneration by clearing debris and suppressing excessive inflammation.

### 3.3 Lipid metabolism in AMD

Lipid dysregulation is a prominent feature of AMD, particularly the dry subtype. Drusen are rich in lipids (up to ~40% by volume), including cholesterol, phosphatidylcholine, and oxidized fatty acid products (51). These deposits arise from both systemic influences (e.g., high plasma lipids) and local retinal dysfunction. The RPE is central to lipid homeostasis in the eye: it phagocytoses the lipid-rich photoreceptor outer segments daily and secretes ApoB-100–containing lipoproteins into Bruch’s membrane as part of a recycling mechanism (52). With age, the RPE’s lipid-processing capacity wanes—phagocytic efficiency declines, and cholesterol-laden debris accumulates in Bruch’s membrane and within RPE cells (as lipofuscin). Drusen are essentially the histological outcome of failed lipid clearance, containing apolipoproteins (B, E), unprocessed cholesterol, and even hydroxyapatite nodules encrusted with lipids and proteins (53).

Microglia play a key role in managing lipid waste in the retina. In the healthy eye, a few microglia patrol the subretinal space, ingesting stray photoreceptor outer segment fragments or other debris not cleared by the RPE. In AMD, as drusen accumulate, microglia are chemotactically drawn to these deposits by danger signals from oxidized lipids (e.g., 7-ketocholesterol, oxidized LDL) (54, 55).

Upon reaching drusen or sites of RPE cell death, microglia attempt to engulf the lipid-rich material. In Cx3cr1-deficient mice (an AMD model), subretinal microglia ingest so much lipid that they transform into grossly engorged “foam cells,” which were initially mistaken for drusen (56). This indicates that without proper regulatory cues, microglia may sequester lipids on-site rather than clearing them. Indeed, foam cell–like microglia are observed in aging human retinas, loaded with intracellular cholesterol crystals and triglycerides. Such lipid-bloated microglia likely become dysfunctional: they adopt a pro-inflammatory phenotype and lose motility, thereby exacerbating drusen accumulation and local inflammation (57).

### 3.4 Neovascularization in AMD

Pathological angiogenesis (choroidal neovascularization) defines wet AMD, and although endothelial cells build the new vessels, immune cells—particularly microglia and macrophages—substantially modulate this process (58). During CNV formation, microglia/macrophages are recruited to neovascular sites by chemokines (e.g., CCL2, CCL5) emanating from ischemic retinal tissue. At the CNV lesions, these myeloid cells can influence angiogenesis through multiple mechanisms (59, 60).

During CNV, multiple retinal myeloid populations accumulate at lesion sites. Available evidence suggests that monocyte-derived macrophages can acquire strongly proangiogenic transcriptional programs, whereas microglia and other myeloid cells may either amplify or restrain lesion growth depending on anatomical niche and timing (31). Myeloid contributions to CNV may include production of inflammatory cytokines, VEGF-family mediators, matrix-remodeling enzymes, and fibrotic signals, as well as recruitment of additional immune cells (38, 61). Conversely, selected retinal myeloid responses may facilitate lesion containment or resolution.

## 4 Targeting microglial TREM2 expression to improve age-related ocular disease outcomes

### 4.1 TREM2: a key microglial receptor for phagocytosis and inflammation

TREM2 is a type I transmembrane receptor of the immunoglobulin superfamily expressed by microglia and other myeloid cells (4). Structurally, it contains an extracellular immunoglobulin-like domain, a stalk region that can undergo proteolytic shedding, a transmembrane domain that associates with TYROBP/DAP12, and a short cytoplasmic tail (62). Because TREM2 lacks an intrinsic signaling motif, ligand binding is transduced through the immunoreceptor tyrosine-based activation motif (ITAM) of DAP12, leading to recruitment of SYK and downstream engagement of PI3K-AKT, PLCγ, ERK/MAPK, and cytoskeletal pathways (63). These signaling events influence migration, phagocytosis, cell survival, metabolic adaptation, and inflammatory calibration.

TREM2 recognizes multiple classes of damage-associated ligands, including anionic lipids, phosphatidylserine exposed on dying cells, lipoproteins, cholesterol-related species, and apolipoproteins such as APOE and APOJ (64). In addition, cleavage of the ectodomain generates soluble TREM2 (sTREM2), which has emerged as a useful pharmacodynamic biomarker in clinical development (65). Functionally, TREM2 is best viewed as a regulator of myeloid state transitions that links damage sensing to phagocytic and immunometabolic responses.

In retinal disease models, Trem2 deficiency impairs myeloid migration and debris handling, whereas microglia-selective Trem2 overexpression or pharmacologic TREM2 agonization can enhance protective responses in selected settings (66–68). However, these effects are not uniformly beneficial across all models or all stages of degeneration. Therefore, TREM2 should be considered a context-dependent regulator of retinal myeloid biology rather than a universally protective switch (69, 70). Conversely, recent retinal degeneration studies suggest that microglia-selective Trem2 overexpression can enhance migratory responses in selected models, whereas other studies demonstrate that Trem2 deficiency worsens photoreceptor degeneration and inflammatory cell recruitment (66, 67).

### 4.2 Therapeutic modulation of TREM2 in microglia and its potential impact on AMD

A surge of recent studies indicates that modulating TREM2 in retinal myeloid cells can alter the course of retinal degeneration. Below, we highlight key findings regarding TREM2 in AMD and related retinal disorders. Collectively, these studies agree that TREM2 expression is invariably altered in age-related retinal disease, although the exact significance of these changes is still debated(Specific study details were shown **Table 1**).

**Table 1:** Targeting TREM2 in AMD-related diseases.

Key factor	Treatments	Experimental models	Methods of administration	Therapeutic effects	Reference
miRNA-34a	Anti-miRNA-34a (AM-34a); PBN, CAPE, CAY10512, or curcumin	Add Aβ42 to C8B4 murine microglial (MG) cells.	NF-kB/miRNA-34a/TREM2	In clinical samples, TREM2 expression is downregulated in human retinal tissues. miR-34a suppresses TREM2 and consequently impairs microglial clearance of Aβ42 in AMD. Treatment with anti–miR-34a or ROS/NF-κB inhibitors restores TREM2 expression toward homeostatic levels and enhances microglial phagocytic capacity.	(11)
Lgals3/Trem2	Tamoxifen	Two-month-old wild-type (WT) mice were used as a young adult baseline. RhoP23H/+ knock-in mice served as a genetic model of photoreceptor degeneration, and sodium iodate (NaIO3) was used to induce acute RPE injury. Two-year-old WT mice were included to model advanced aging.	Trem2/Gal3/subretinal microglia phagocytosis	Subretinal microglia impede disease progression via the Gal3–TREM2 signaling pathway, and this response can be enhanced pharmacologically.	(10)
IFN-I	Tamoxifen	Mice homozygous for*Pde6b* mutation (rd10 mice) (Gempharmatech Co), *Trem2* knockout (*Trem2*^−/−^) mice, *Tmem119^CreERT2^* mice (*CreERT2* was inserted at the end of exon 2), *Rosa26^CAG − LSL−Trem2^* (*Trem2*-transgenic).*Trem2*^−/−^ mice were crossed with rd10 mice to obtain *Trem2*-deficient rd10 mice (*Trem2^−/−^*:rd10). *Tmem119^CreERT2^* mice were crossed with *Rosa26^CAG − LSL−Trem2^* mice to obtain microglia-specific *Trem2* overexpression mice(*Tmem119^CreERT2^*:*Rosa26^CAG − LSL−Trem2^*) To induce retinal degeneration in mice, 60 mg/kg BW of MNU was administrated by intraperitoneal injection.	Trem2/IFN-I signaling/microglial migration-phagocytosis	Trem2-mediated microglial migration and subsequent phagocytosis exert divergent effects on cell death at different stages of the RP-featured photoreceptor degeneration model. And IFN-I blockade suppressed migratory response of retinal microglia with Trem2 overexpression.	(67)
SOCS3	Genetic Trem2 haploinsufficiency and myeloid-specific Socs3 deficiency	C57BL/6J mice, LysM‐Cre mice, Ai9 flox mice, and *Trem2* knockout mice. *Socs3* flox (*Socs3 ^f/f^*^)^ mice were crossed with LysM‐Cre mice to generate myeloid‐specific *Socs3* knockout mice (*Socs3* ^cKO^), which were then crossed with Trem2 knockout mice to generate compound mutants. Choroidal neovascularization was induced using the Micron IV Image‐Guided Laser System	Trem2/SOCS3/angiogenic-inflammasome programs	TREM2-expressing myeloid cells limit CNV lesion size and vascular leakage. Trem2 and SOCS3 define overlapping but independent anti-angiogenic programs, and combined deficiency additively worsens CNV pathology.	(71)
PPARγ/CD36	GW0742, a PPARβ/δ agonist, and GW1929, a PPARγ agonist	MNU-induced retinal degeneration in WT and Trem2^-/-^ mice.	TREM2/PPARγ/CD36	Trem2 deficiency deterred microglial infiltration, exacerbated photoreceptor cell death, and aggravated retinal inflammation during retinal degeneration.And PPARγ and CD36 are important effector molecules of TREM2, contributing to the protective function of activated microglial cells during photoreceptor cell degeneration	(70)
Trem2	Purified bovine sulfatide and agonist anti-TREM2 antibody	Laser-induced CNV in WT and Trem2^-/-^ mice, plus RAW264.7 macrophages *in vitro*	TREM2/TNF/phagocytosis	Pharmaceutical activation of TREM2 suppressed CNV formation *in vivo* associated with reduction of Tnf and promotion of phagocytosis	(72)

Bhattacharjee (11) et al. reported that retinal TREM2 expression declines with age, especially in AMD patients, correlating with an upregulation of miR-34a. *In vitro*, treating microglial cultures with antioxidants, NF-κB inhibitors, or miR-34a inhibitors restored TREM2 levels and enhanced microglial phagocytosis of Aβ-42, suggesting that oxidative stress and specific microRNAs can downregulate TREM2 during aging (11).

One of the main causes of vision loss in AMD is the atrophy of photoreceptors and the RPE. Yu (10) et al. found that the Trem2 signaling pathway regulates microglial migration to areas of atrophy and induces galectin-3 expression. In conditional Trem2 knockout mice, retinal degenerative lesions were exacerbated, with fewer microglia and reduced galectin-3 expression; pharmacologically increasing Trem2 expression reversed this trend. He (67) et al. also found that Trem2 knockout mice were more prone to retinal degenerative changes, specifically showing increased photoreceptor cell death. Type I interferon signaling is closely tied to microglial migration and is modulated by TREM2 expression. Zhou et al. (70) similarly observed that mice with Trem2 specifically knocked out exhibited more severe photoreceptor cell death and reduced microglial migratory capacity, along with earlier thinning of the outer nuclear layer, with a mechanism involving the PPARγ pathway and CD36.

Wang et al. (71) observed that in a wet AMD model, microglial TREM2 expression increased after CNV onset. Mice with Trem2 knockdown developed larger CNV lesions and more severe vascular leakage, indicating that TREM2 restrains pathological angiogenesis, likely by modulating inflammatory and angiogenic signaling. Likewise, another group reported that enhancing Trem2 expression in mononuclear phagocytes suppressed choroidal neovascular growth and ameliorated disease severity (72).

Some research suggests that loss of the TREM2-DAP12 pathway causes microglial dysfunction, upregulating STAT3-related pathways and reducing the ability of microglia to phagocytose apoptotic cells and myelin, thereby exacerbating neurodegenerative disease symptoms (73). However, differences exist in retinal pathology: under hypoxic conditions, Müller glia can be activated by TREM2/DAP12, which in concert with CSF1R activates spleen tyrosine kinase (SYK), subsequently promoting HIF1α expression and high levels of angiogenic factors like VEGF, exacerbating retinal vascular lesions (74).

**Figure 2:**
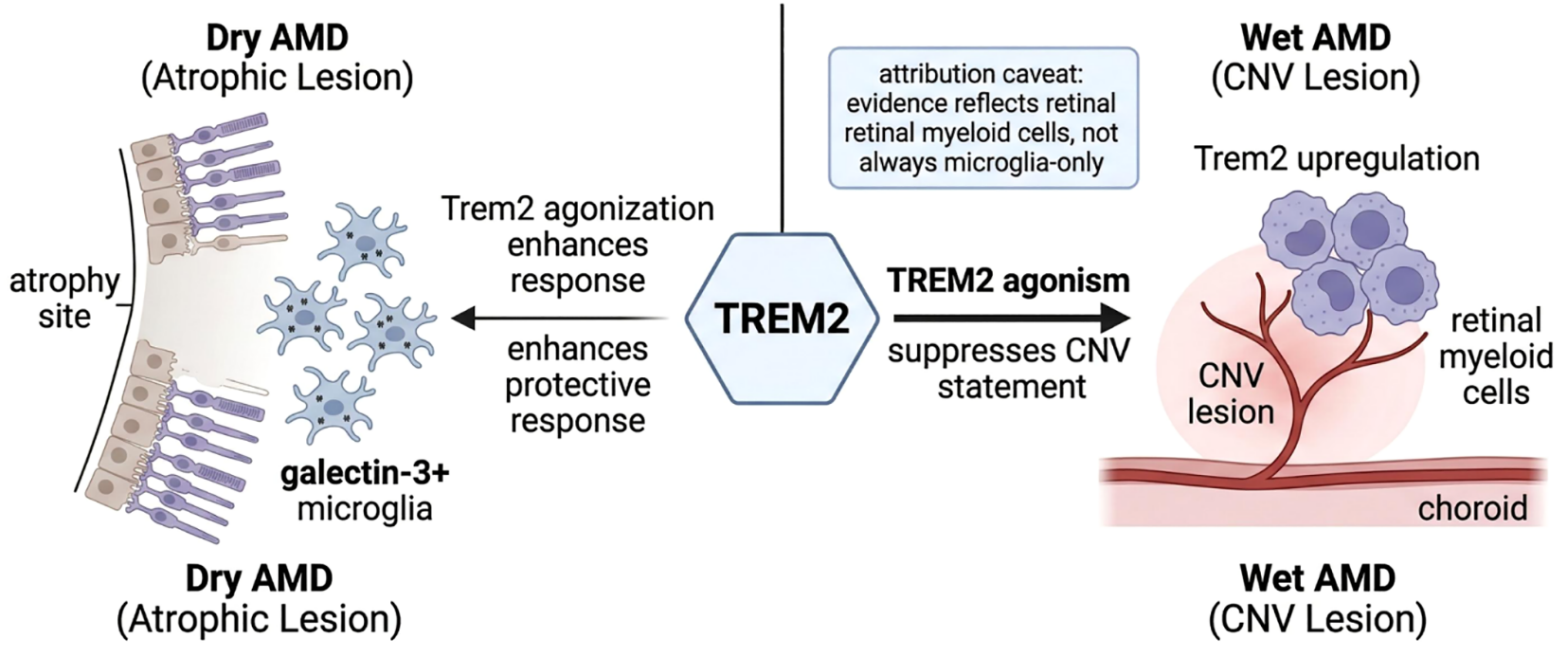
Schematic diagram depicting the beneficial role of Trem2 in microglia during AMD disease progression. (In dry AMD, retinal TREM2 is reduced with aging and AMD, whereas TREM2 activation promotes galectin-3+ microglial migration to atrophic lesions and supports protective responses against photoreceptor degeneration. In wet AMD, TREM2 is upregulated in lesion-associated retinal myeloid cells after CNV onset, and enhancing TREM2 signaling suppresses neovascular growth and vascular leakage).

Additional findings underscore the interplay between TREM2 and other factors in AMD. One study of a high-risk complement factor H (CFH) variant showed that this mutation accelerates microglial accumulation and heightens oxidative/inflammatory stress in the retina; interestingly, aged CFH transgenic mice exhibited elevated Trem2 expression, potentially as a compensatory response to chronic inflammation (75). Links between AMD and Alzheimer’s disease have also emerged: β-amyloid deposits were detected in periocular drainage systems of AD patients, suggesting that CNS amyloid can reach the eye via the optic nerve and contribute to retinal degeneration (76). Shi (77) et al. found that inhibiting miR-155 in the TREM2-APOE pathway effectively reduced the Clec7a^+^ and galectin-3^+^ microglial populations, restored microglial homeostasis, and improved the tight junctions of the perivascular blood–retinal barrier(A schematic diagram is shown in **Figure 2**).

## 5 Discussion

Taken together, current evidence supports a retinal myeloid landscape model rather than a strictly microglia-only model of AMD. In atrophic degeneration, TREM2-associated programs appear to facilitate migration to lesion sites, induction of galectin-3-positive protective states, lipid and debris clearance, and preservation of outer retinal structure (23, 24). In neovascular disease, TREM2 modulation may reduce CNV severity, but most currently available studies manipulate retinal myeloid cells broadly through pharmacologic agonists, global knockouts, or lesion-wide readouts. Therefore, the present evidence supports TREM2 as a promising retinal myeloid target, but not yet as an exclusively microglial or uniformly beneficial pathway across all AMD stages.

Current evidence suggests that enhancing the TREM2 signaling pathway has potential therapeutic value in AMD. In atrophic models of dry AMD, lack of TREM2 leads to impaired microglial recruitment and clearance of dead photoreceptors, thereby exacerbating retinal degeneration. This indicates that TREM2 is required to initiate an effective, protective DAM response characterized by enhanced phagocytic capacity and suppression of excessive inflammation (44). Similarly, in CNV models of wet AMD, TREM2 activation appears to inhibit pathological neovascular growth by modulating inflammation and angiogenic signals, thus providing protection (29). It should be emphasized that despite a substantial body of evidence supporting a protective role for TREM2 in AMD, details of TREM2’s function and its stage-specific effects remain controversial. Some studies suggest that the impact of microglia on retinal degeneration may be biphasic: at different disease stages or in different microenvironments, microglia might be “helpers” in some contexts but become “accomplices” in others. The role of TREM2 may also evolve with disease progression (78). Moderate TREM2-mediated activation in early AMD helps with debris clearance and inflammation limitation, but in later stages it is unclear if sustained high TREM2 expression is always beneficial. One study noted that loss of the TREM2/DAP12 pathway disrupts microglial function, exacerbating neurodegenerative damage (73); however, in the retina, TREM2 signaling has also been linked to certain pro-pathogenic pathways, such as potentially promoting VEGF expression under hypoxic conditions and worsening vascular abnormalities (79). These seemingly contradictory findings suggest that the “double-edged sword” effect of TREM2 activity needs to be carefully weighed according to disease stage and microenvironment. Additionally, different immune cell subtypes (resident microglia vs. infiltrating macrophages) have distinct roles in AMD and may respond differently to TREM2 signaling, which remains an unresolved question in current research.

Another caveat is that our current understanding of TREM2 in AMD largely stems from animal studies and *in vitro* systems. The exact mechanisms by which TREM2 modulates microglial function in the human retina remain incompletely defined. We still do not fully understand why TREM2 expression is reduced in AMD patients’ retinas, nor what repertoire of ligands drive TREM2 signaling in the ocular milieu (candidates include various phospholipids, apolipoproteins, amyloid fragments, etc.). Further in-depth studies on retinal TREM2 pathways and targets in human tissue are needed and will be crucial for devising precise therapeutic strategies.

On the therapeutic front, tangible strategies to boost TREM2 signaling in the retina are still in development. Possible approaches include gene therapies to elevate TREM2 expression, agonistic antibodies or small molecules to activate TREM2, and inhibitors of negative regulators of TREM2. Importantly, any such intervention must be finely calibrated: the timing and dosage of TREM2 enhancement will be critical, as overly vigorous microglial activation could provoke harmful inflammation. The goal is to restore microglial homeostasis and protective functions without inciting collateral damage—a balance that future research must address.

TREM2 agonist programs in neurodegeneration provide translational lessons for AMD. Several TREM2 agonist modalities (agonistic antibodies and small molecules) have progressed into human studies, with clinicaltrials.gov records offering the most reliable up-to-date status. For example, the agonistic anti-TREM2 antibody AL002 was evaluated in early Alzheimer’s disease in a randomized phase 2 study (INVOKE−2; NCT04592874), which has completed and was publicly reported by the sponsor as not meeting its primary clinical endpoint despite pharmacodynamic engagement (80). In parallel, the oral small-molecule TREM2 agonist VG−3927 has been registered in a first-in-human phase 1 study to assess safety/PK/PD, illustrating a non-antibody approach to TREM2 activation (81). A separate agonistic antibody program VGL101/iluzanebart has been studied in ALSP (NCT05677659), highlighting interest in TREM2 activation across neurodegenerative indications (64). Collectively, these programs show that systemic target engagement is feasible, but also caution that clinical benefit likely depends on patient selection, disease stage, and biomarker strategy. For AMD, translation may benefit from the eye’s local delivery options (intravitreal/subretinal) but will require careful evaluation of cell-type specificity (microglia vs monocyte-derived macrophages), lesion stage (atrophy vs CNV/fibrosis), and ocular pharmacodynamic readouts.

Outstanding questions and future directions. Four priorities emerge for the field: (1) Cell-of-origin assignment—quantify the relative contributions of resident microglia versus recruited monocyte-derived macrophages to TREM2-dependent phenotypes in human dry AMD versus nAMD/CNV (ideally via spatial transcriptomics and fate-mapping-informed markers); (2) Niche and stage dependency—determine whether TREM2 agonism should be timed to early debris clearance/lesion restriction versus later chronic inflammation/fibrosis; (3) Ocular pharmacodynamic biomarkers—develop aqueous/vitreous readouts (e.g., sTREM2 and inflammatory mediators) and imaging correlates to confirm target engagement in the eye; (4) Combination strategies and safety—test whether TREM2 modulation can synergize with anti-VEGF and/or complement-directed therapies while monitoring risks such as excessive inflammation, fibrosis, or off-target activation of peripheral myeloid compartments.

In conclusion, despite the unanswered questions, targeting microglial TREM2 holds significant promise as a novel therapeutic strategy for AMD. Ongoing research into TREM2’s functions and regulatory networks will help close current knowledge gaps and propel the development of more effective, multifaceted interventions to prevent and treat AMD.
